# Human Herpesvirus 6 Infection Presenting as an Acute Febrile Illness Associated with Thrombocytopenia and Leukopenia

**DOI:** 10.1155/2016/2483183

**Published:** 2016-11-17

**Authors:** Maja Arnež, Tatjana Avšič-Županc, Tina Uršič, Miroslav Petrovec

**Affiliations:** ^1^University Medical Center Ljubljana, Japljeva 2, SI-1525 Ljubljana, Slovenia; ^2^Institute of Microbiology and Immunology, Faculty of Medicine, University of Ljubljana, Zaloška 4, SI-1000 Ljubljana, Slovenia

## Abstract

We present an infant with acute fever, thrombocytopenia, and leukopenia, coming from an endemic region for tick-borne encephalitis, human granulocytic anaplasmosis, and hantavirus infection. The primary human herpesvirus 6 infection was diagnosed by seroconversion of specific IgM and IgG and by identification of viral DNA in the acute patient's serum. The patient did not show skin rash suggestive of exanthema subitum during the course of illness.

## 1. Introduction

Slovenia, a Central European country, is an endemic region for at least three zoonotic infectious diseases that may present as an acute febrile illness associated with thrombocytopenia and leukopenia: tick-borne encephalitis (TBE), human granulocytic anaplasmosis, and hantavirus infection [1, 2].

We present a patient with exanthema subitum without exanthema, mimicking the three zoonotic infectious illnesses mentioned above.

## 2. Case Presentation

In September 2015, an eleven-month-old girl was referred to our department with a 2-day history of high fever without other symptoms. Two days before she got ill, she had been in direct contact with mouse stool. Epidemiological data on previous tick bite was negative. On physical examination, she was febrile (38.9°C) and no other clinical signs were found. Initial laboratory findings revealed thrombocytopenia, leukopenia, and severe absolute neutropenia (Table 1). She was treated symptomatically as outpatient.

We searched for bacteremia, respiratory viruses, TBE virus,* Anaplasma phagocytophilum*, and hantaviruses. Within the next two days, a rapid drop in body temperature to normal was observed. She had no symptoms and no signs of illness thereafter. Since clinical picture and laboratory results were nonspecific, the diagnosis of exanthema subitum was confirmed by microbiological investigations (Table 2).

## 3. Discussion

HHV-6 is a member of the* Herpesvirales* order, Herpesviridae family,* Betaherpesvirinae* subfamily, and* Roseolovirus* genus. HHV-6A and HHV-6B are two distinct species of HHV-6 [3].

Primary infection with HHV-6 had been shown to be the cause of exanthema subitum (roseola) in infants and can also result in an infectious mononucleosis-like illness in adults [4]. Serologic studies indicate that most people become infected by the age of two, most likely through saliva transmission [3, 4]. Following its entry into the body by the oral route, the virus replicates in the salivary glands and satellite lymphoid tissues of the oropharynx, probably the tonsils and cervical lymph nodes. Systemic dissemination is the result of viremia [3].

Exanthema subitum is characterized by an abrupt rise in body temperature to as high as 40°C followed by a rapid drop to normal within the next 2–4 days which coincides with the appearance of an erythematous maculopapular skin rash that persists for 1–3 days [5]. But only 17% of infants and young children with primary HHV-6 infection have a rash, clinically compatible with exanthema subitum [6]. Primary HHV-6 infection is a common benign and self-limiting illness of infancy [7]. However, primary HHV-6 infection can cause acute encephalopathy without exanthema [8]. HHV-6 is sometimes the etiological agent of febrile seizures [5]. Thrombocytopenia accompanied by neutropenia and leukopenia during the acute phase of exanthema subitum, serologically confirmed as primary HHV-6 infection, has been reported in the literature [7]. These laboratory changes were found in children with 9–14 months of age and were self-limiting without serious complications. All patients had a skin rash. Thrombocytopenia was the result of bone marrow suppression rather than of immune-mediated peripheral consumption seen in acute idiopathic thrombocytopenic purpura or from disseminated intravascular coagulation [7].

The diagnosis of exanthema subitum is usually clinical. In atypical cases, the diagnosis of primary HHV-6 infection is performed by both serologic and direct methods [3]. For patients with primary infection, serological studies show the appearance of specific IgM antibodies during the first week and their subsequent disappearance after month, while IgG antibodies are detected later than IgM but persist indefinitely. Direct detection of HHV-6 by PCR in blood of patient with primary infection is also possible during the febrile phase of illness [3]. When the patient shows no skin rash, the etiological diagnosis is possible only by examining the blood [8]. Reactivation or reinfection with HHV-6 may also occur in an individual who is already seropositive [3].

No therapies are approved currently for the treatment of HHV-6 infection [4]. Intravenous ganciclovir and foscarnet are proposed as first-line drugs for HHV-6 encephalitis for 3-4 weeks [3].

We present the eleven-month-old girl with exanthema subitum with an acute fever, thrombocytopenia, leukopenia, and neutropenia but without skin rash suggestive of primary HHV-6 infection (Table 1). A careful review of the literature revealed no similar case of exanthema subitum; however, thrombocytopenia is reported to be a complication of exanthema subitum [7]. Patient with acute encephalopathy with primary HHV-6 infection with no skin rash has also been described [8]. Many infectious diseases can cause acute febrile illness associated with thrombocytopenia and leukopenia. In our patient microbiological testing for influenza A and B, parainfluenza virus, respiratory syncytial virus, rhinovirus, adenovirus, enterovirus, metapneumovirus, and human bocavirus by PCR from nasopharyngeal swab in the acute illness was negative. We also excluded serious bacterial infection and acute infection with TBE virus, cytomegalovirus (CMV), hantaviruses, and* Anaplasma phagocytophilum*. It is important to differentiate these infections because therapeutic approaches differ considerably. Acute HHV-6 infection in our patient was established by seroconversion of IgM and IgG antibodies against HHV-6 and directly by demonstration of DNA-HHV-6 in patient's blood by PCR (Table 2). HHV-6 is spreading by blood to different organs [3]. In our patient, it spread from blood to bone marrow and caused laboratory abnormalities, but obviously it did not spread to skin as would be expected in typical exanthema subitum.

In conclusion, primary HHV-6 infection in infancy can present as an acute febrile illness associated with thrombocytopenia, leukopenia, and neutropenia but without skin rash suggestive of exanthema subitum.

## Figures and Tables

**Table 1:** Laboratory findings of patient with exanthema subitum without exanthema presented as an acute febrile illness associated with thrombocytopenia and leukopenia.

Variable (normal values)	Acute illness (20/09/2015–23/09/2015)	Convalescent illness (23/09/2015–02/10/2015)
C-reactive protein (<5 mg/L)	<5	<5
Leukocytes (4.0–10.0 × 10^9^/L)	3.0	8.9
Absolute neutrophil counts (1.6–7.5 × 10^9^/L)	0.4	2.8
Erythrocytes (4.5–6.3 × 10^12^/L)	5.05	5.06
Platelets (140–340 × 10^9^/L)	76	261
Bilirubin (≤17 *μ*mol/L)	6	7
Alkaline phosphatase (2.07–5.69 *μ*kat/L)	3.96	4.21
Alanine aminotransferase (≤0.60 *μ*kat/L)	0.35	0.36
Aspartate aminotransferase (≤0.86 *μ*kat/L)	1.27	0.75
Gamma glutamyl transpeptidase (0.02–0.65 *μ*kat/L)	0.18	0.17

**Table 2:** Microbiological findings in blood of patient with exanthema subitum without exanthema presented as an acute febrile illness associated with thrombocytopenia and leukopenia.

Variable (date)	Acute illness (20/09/2015–23/09/2015)	Convalescent illness (23/09/2015–02/10/2015)
Blood culture	Negative	ND
PCR-DNA-HHV-6	Positive	ND
IFA-IgM HHV-6	Negative	Positive
IFA-IgG HHV-6	Negative	Positive
RT-PCR-RNA-TBE virus	Negative	ND
ELISA-IgM TBE virus	Negative	Negative
ELISA-IgG TBE virus	Negative	Negative
PCR-DNA *Anaplasma phagocytophilum*	Negative	ND
IFA-IgG* Anaplasma phagocytophilum*	Negative	Negative
RT-PCR-RNA hanta virus	Negative	ND
IFA-IgG Hantaan, Puumala, Dobrava	Negative	Negative
ELISA-IgM Hantaan, Puumala	Negative	Negative
ELISA-IgG Hantaan, Puumala, Dobrava	Negative	Negative
CLIA-IgM CMV	Negative	Negative
CLIA-IgG CMV	Positive	Positive

ND, not done; PCR, polymerase chain reaction; RT-PCR, real-time polymerase chain reaction; IFA, immunofluorescent assay; ELISA, enzyme linked immunosorbent assay; CLIA, chemiluminescence immune assay; HHV-6, human herpesvirus 6; TBE, tick-borne encephalitis; CMV, cytomegalovirus.

## Abbreviations

HHV-6: Human herpesvirus 6
PCR: Polymerase chain reaction
TBE: Tick-borne encephalitis.
